# Frequency and risk factors for rebleeding events in patients with small bowel angioectasia

**DOI:** 10.1186/s12876-014-0200-3

**Published:** 2014-11-28

**Authors:** Eiji Sakai, Hiroki Endo, Masataka Taguri, Harunobu Kawamura, Leo Taniguchi, Yasuo Hata, Akiko Ezuka, Hajime Nagase, Takaomi Kessoku, Ken Ishii, Jun Arimoto, Eiji Yamada, Hidenori Ohkubo, Takuma Higurashi, Tomoko Koide, Takashi Nonaka, Hirokazu Takahashi, Atsushi Nakajima

**Affiliations:** Department of Gastroenterology and Hepatology, Yokohama City University School of Medicine, 3-9 Fuku-ura, Kanazawa-ku, Yokohama, 236-0004 Japan; Department of Endoscopy Center, Yokohama City University School of Medicine, Yokohama, Japan; Department of Biostatistics and Epidemiology, Yokohama City University School of Medicine, Yokohama, Japan; Gastroenterology Division, Odawara Municipal Hospital, Odawara, Japan; Gastroenterology Division, Chigasaki Municipal Hospital, Chigasaki, Japan; Gastroenterology Division, Yokohama Rosai Hospital, Yokohama, Japan; Gastroenterology Division, Hiratsuka City Hospital, Hiratsuka, Japan

**Keywords:** Capsule endoscopy (CE), Balloon-assisted endoscopy (BAE), Small bowel angioectasia, Obscure gastrointestinal bleeding (OGIB), Argon plasma coagulation (APC)

## Abstract

**Background:**

Small bowel angioectasia is reported as the most common cause of bleeding in patients with obscure gastrointestinal bleeding. Although the safety and efficacy of endoscopic treatment have been demonstrated, rebleeding rates are relatively high. To establish therapeutic and follow-up guidelines, we investigated the long-term outcomes and clinical predictors of rebleeding in patients with small bowel angioectasia.

**Methods:**

A total of 68 patients were retrospectively included in this study. All the patients had undergone CE examination, and subsequent control of bleeding, where needed, was accomplished by endoscopic argon plasma coagulation. Based on the follow-up data, the rebleeding rate was compared between patients who had/had not undergone endoscopic treatment. Multivariate analysis was performed using Cox proportional hazard regression model to identify the predictors of rebleeding. We defined the OGIB as controlled if there was no further overt bleeding within 6 months and the hemoglobin level had not fallen below 10 g/dl by the time of the final examination.

**Results:**

The overall rebleeding rate over a median follow-up duration of 30.5 months (interquartile range 16.5–47.0) was 33.8% (23/68 cases). The cumulative risk of rebleeding tended to be lower in the patients who had undergone endoscopic treatment than in those who had not undergone endoscopic treatment, however, the difference did not reach statistical significance (*P* = 0.14). In the majority of patients with rebleeding (18/23, 78.3%), the bleeding was controlled by the end of the follow-up period. Multiple regression analysis identified presence of multiple lesions (≥3) (OR 3.82; 95% CI 1.30–11.3, *P* = 0.02) as the only significant independent predictor of rebleeding.

**Conclusion:**

In most cases, bleeding can be controlled by repeated endoscopic treatment. Careful follow-up is needed for patients with multiple lesions, presence of which is considered as a significant risk factor for rebleeding.

## Background

Obscure gastrointestinal bleeding (OGIB), defined as persistent or recurrent bleeding with negative findings on upper and lower gastrointestinal endoscopic evaluations [1], accounts for about 5% of all cases of gastrointestinal bleeding [2]. Capsule endoscopy (CE), introduced in 2000, has become established as the examination modality of first choice for the investigation of OGIB [3-6] and other small bowel abnormalities [7-10]. The diagnostic yield of CE has been reported to range from 45% to 80% [6,11-15], as high as that of balloon-assisted enteroscopy (BAE) [16,17].

Angioectasia is the most commonly occurring vascular malformation of the gastrointestinal tract [18]. Although small bowel angioectasia was previously considered to be rare, with recent advances in endoscopic modalities (e.g. CE and BAE), this condition is being detected increasing frequently in clinical practice. Recent studies have revealed that small bowel angioectasia is the most common cause of bleeding, sometimes life-threatening, in patients with OGIB [19,20]. Although several studies have demonstrated the safety and efficacy of endoscopic treatment for small bowel angioectasia [21,22], not all patients with OGIB can receive endoscopic treatment, because the procedure is complex and time-consuming. Moreover, relatively high rebleeding rates have been reported in patients with small bowel angioectasia [23-26] and the predictors of rebleeding have not yet been fully clarified. Therefore, there is a need for therapeutic and follow-up guidelines to be established.

We conducted the present study in a relatively large cohort to determine the efficacy of endoscopic treatment and the long-term outcomes in patients with small bowel angioectasia. In addition, the clinical predictors of rebleeding were evaluated to identify patients who would need close follow-up.

## Methods

### Patients

This cohort study was conducted to reveal the long-term outcomes in patients with small bowel angioectasia. Of 386 consecutive patients with OGIB who underwent CE at Yokohama City University Hospital or any of four tertiary hospitals (Yokohama Rosai Hospital, Chigasaki Municipal Hospital, Odawara Municipal Hospital and Hiratsuka City Hospital) between October 2007 and October 2012, we enrolled 74 patients who were detected to have at least one small bowel angioectasia. All of the patients had undergone upper and lower endoscopic examinations prior to the CE, with negative findings. To investigate the long-term outcomes of patients with small bowel angioectasia, and the risk factors for rebleeding in these patients, patients with other definitive lesions (e.g. ulcers, Dieulafoy’s lesions, varices, anteriovenous malformations, diverticula or tumors) were excluded. In addition, patients with failed CE examination due to CE retention, incomplete small bowel transit or poor bowel preparation were also excluded. The study protocol was approved by the Ethics Committee of Yokohama City University Hospital, Odawara Municipal Hospital, Chigasaki Municipal Hospital, Yokohama Rosai Hospital and Hiratsuka City Hospital. Written informed consent was obtained from all of the subjects prior to their participation in the study.

### Clinical information

We registered the patient data from the database, including the type of OGIB, the age and sex of the patients, smoking history, alcohol history, blood transfusion history, minimum hemoglobin concentration, presence/absence of comorbidities, and the current medication history at the time of the initial CE. According to the bleeding pattern, the OGIB was classified into two categories; overt, manifesting as melena or hematochezia, and occult, manifesting as recurrent IDA and/or a positive fecal occult blood test without any visible bleeding. In addition to the information from the database, follow-up data, including the data at the final examination, change in the hemoglobin level, presence/absence of overt bleeding, and the treatment history were obtained retrospectively from the hospital medical records or the responses to questionnaires collected by the doctors of other hospitals/clinics. The follow-up duration was defined as the time from the first CE examination to the last medical examination.

### Capsule endoscopy

The patients were instructed to swallow the CE capsule (PillCam SB or SB2; Given Imaging, Yoqneam, Israel) with a solution of dimethicone after fasting overnight, with no other bowel preparation. They were allowed to drink clear liquids 2 hours after they had swallowed the capsule, and a light meal 4 hours after. Two CE experts (with experience of reporting more than 150 CE videos) separately read and interpreted the complete CE videos. When there were discrepancies in the interpretation, both the experts reviewed the findings simultaneously and reached a consensus.

### Definition of small bowel angioectasia

**Figure 1:**
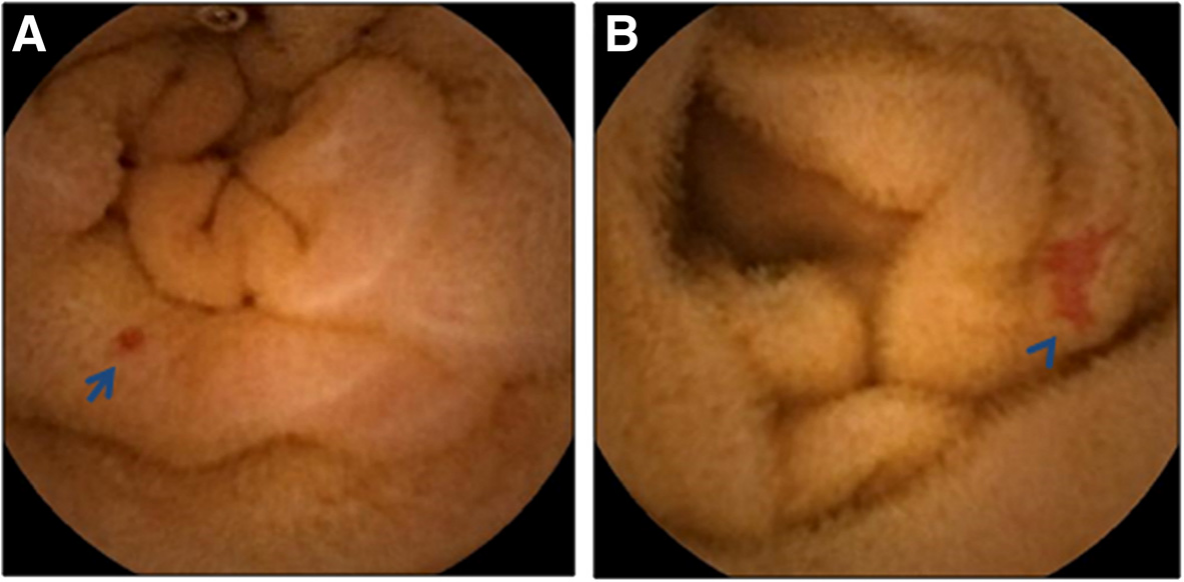
**Capsule endoscopic findings of small bowel angioectasia. A**: punctate angioectasia (arrow). **B**: patchy angioectasia (arrowhead).

Angioectasia is a venous lesion that requires cauterization; Dieulafoy's lesions and arteriovenous malformations may cause arterial bleeding, and require clipping or surgical treatment. According to a previous report [27], angioectasia is a punctate (<1 mm) or patchy (a few mm) erythematous lesion (Figure 1) with or without oozing, that is diagnosed by CE and/or BAE, as histologic confirmation cannot be obtained for most of these lesions. In the present study, both punctate and patchy erythema were considered as definitive diagnostic findings, and the locations and sizes of the angioectasia were recorded according to the results of the CE examination. Each of the CE videos was divided into two segments of equal length according to the small-bowel transit time; the first segment was considered as representing the proximal small bowel and the second as representing the distal small bowel.

### Treatment of small bowel angioectasia

In this study, all the patients had undergone CE examination prior to any endoscopic treatment. Subsequent endoscopic treatment was undertaken when active bleeding was identified by CE. In addition, endoscopic treatment was also undertaken for patients with ongoing overt bleeding (within three days) and/or drop of the hemoglobin level by more than 2 g/dl within two weeks after the first episode of OGIB. If endoscopic treatment was needed to control small bowel bleeding, single-balloon enteroscopy (SBE) (SIF-Q260, Olympus Optical, Tokyo, Japan) was performed within 5 days of the initial CE examination. The insertion route was determined by the location of the lesions detected by the CE. If a definitive bleeding source could not be identified by one route, another route was used. For cases where a bleeding source was clearly detected during the procedures, total enteroscopy was not always attempted. For angioectasias, endoscopic argon plasma coagulation (APC) was performed at an output of 40 W and an argon gas flow rate of 1.6 L/min. Basically, an attempt was made to treat all the angioectasias detected by the SBE, although some non-bleeding small angioectasias were left untreated if there were too many lesions to treat. No serious complications were encountered in any of the patients during the study.

### Definition of the rebleeding and control rate

The main outcome variable of this study was the incidence of recurrent bleeding. Rebleeding was defined as evidence of recurrent visible gastrointestinal bleeding (hematochezia or melena) with recent negative upper and lower endoscopic examinations and/or a reccurent drop of the hemoglobin level by more than 2 g/dl from the baseline. We defined the OGIB as controlled when there was no further overt bleeding within 6 months of the initial episode and the hemoglobin level did not drop to below 10 g/dl by the time of the final examination.

### Statistical analysis

The statistical significances of differences in the values of the clinical parameters were evaluated by Fisher’s exact test and an unpaired student’s *t*-test. Follow-up data related to the rebleeding-free interval were analyzed by the Kaplan-Meier method and log-rank test. Univariate and multivariate analyses were performed using Cox proportional hazard regression models to identify the predictors of rebleeding in patients with small bowel angioectasia. For the multivariate analysis, only variables identified as being significant with *P* values of <0.1 by the univariate analyses were included as covariates. Unless otherwise specified, *P* values of <0.05 were considered to denote statistical significance. All the analyses were performed using SPSS, ver. 11.0 (SPSS Inc., Chicago IL, USA).

## Results

### Patients

**Figure 2:**
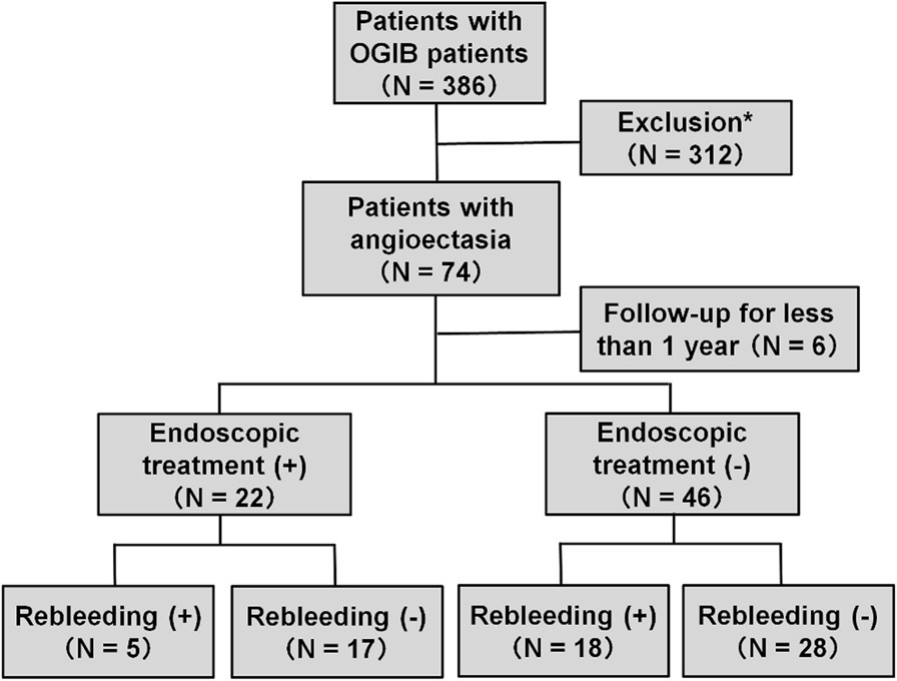
Study flow diagram. *Patients with other definitive small bowel lesions, such as ulcers, Dieulafoy’s lesions, varices, anteriovenous malformations, diverticula and tumors, were excluded.

Of the 74 patients enrolled in this study, 6 patients who were followed up for less than 1 year, were lost to follow-up, or died of other causes were excluded. Finally, a total of 68 patients were included for the analysis in this study (Figure 2).

### Demographic characteristics and clinical data

The demographic and clinical characteristics of the patients with small bowel angioectasia are shown in Table 1. Of the 68 patients, 22 (32.4%) received endoscopic treatment, while 46 (67.6%) were managed conservatively with or without iron replacement therapy. While 17 patients who underwent endoscopic treatment (77.3%) required blood transfusion, only 14 patients who did not undergo endoscopic treatment (30.4%) needed blood transfusion (*P* <0.001). The minimum hemoglobin level in the patients who had undergone endoscopic treatment was significantly lower than that in the patients who had not undergone endoscopic treatment (8.0 ± 2.0 vs. 9.9 ± 2.9, *P* = 0.006). There was no significant difference in the prevalence of comorbidities, except for that of hypertension (86.4% vs. 58.7%, *P* = 0.03), or the rate of medication use between the patients who had/had not undergone endoscopic treatment.

**Table 1:** **Demographic and clinical characteristics of the study patients**

**Characteristics**	**Total**	**Endoscopic treatment (+)**	**Endoscopic treatment (-)**	***P*** value*
Number	68	22	46	
Bleeding pattern (overt/occult)	40/28	19/3	21/25	0.002
Number of angioectasia, mean (median)	3.4 (2.5)	4.0 (2.0)	3.1 (3.0)	0.68
Age, year, mean ± SD	67.6 ± 12.8	66.9 ± 10.9	66.5 ± 13.6	0.30
Sex, Male/Female	38/30	11/11	27/19	0.60
Drinking history (%)	23 (33.8)	11 (50.0)	12 (26.0)	0.06
Smoking history (%)	24 (35.3)	7 (31.8)	17 (37.0)	0.79
Blood transfusion (%)	31 (45.6)	17 (77.3)	14 (30.4)	0.001
Minimum hemoglobin value, g/dl	9.3 ± 2.7	8.0 ± 2.0	9.9 ± 2.9	0.006
Follow-up duration, month, median (IQR)	30.5 (16.5-47.0)	34.0 (21.0-46.5)	30.0 (18.0-46.0)	0.76
Rebleeding rate, number (%)	23 (33.8)	5 (22.7)	18 (39.1)	0.27
Iron replacement therapy after OGIB, number (%)	59 (86.8)	20 (91.0)	39 (84.8)	0.71
Comorbidity, number (%)				
Hypertension	46 (67.6)	19 (86.4)	27 (58.7)	0.03
Diabetes	15 (22.1)	7 (31.8)	8 (17.4)	0.22
Cardiovascular disease	18 (26.5)	8 (36.4)	10 (21.7)	0.25
Cerebral infarction	9 (13.2)	3 (13.6)	6 (13.0)	>0.99
CKD, ≥ stage 4	17 (86.4)	8 (36.4)	9 (19.6)	0.15
Liver cirrhosis	3 (4.4)	1 (4.5)	2 (4.3)	>0.99
Medication used, number (%)				
Warfarin	9 (13.2)	4 (18.2)	5 (10.9)	0.46
LDA	27 (39.7)	10 (45.5)	17 (37.0)	0.60
Thienopyridine	9 (13.2)	3 (13.6)	6 (13.0)	>0.99
NSAIDs	7 (10.3)	2 (9.1)	5 (10.9)	>0.99
H2-blockers	17 (25.0)	5 (22.7)	12 (26.1)	>0.99
PPIs	25 (36.8)	8 (36.4)	17 (37.0)	>0.99
Rebamipide	10 (14.7)	4 (18.2)	6 (13.0)	>0.99

*Abbreviations*: *IQR*, interquartile range; *CKD*, chronic kidney disease; *LDA*, low-dose aspirin; *NSAIDs*, nonsteroidal anti-inflammatory drugs; *H2-blockers*, histamine H2 receptor antagonists; *PPIs*, proton pump inhibitors.

Variable definitions: Alcohol history was defined as positive if the subject’s alcohol consumption exceeded 20 g/day. Smoking history was defined as positive if the subject had smoked more than 10-pack years and was still smoking or had quit within the previous 10 years. History of antiplatelet drug and/or NSAID use was defined as positive if the patient had been taking at least 1 pill per day of either of these drugs for more than 1 week within 1 month prior to the CE. History of anticoagulant drug use was defined as positive if the patient had been taking at least 1 pill of anticoagulant drug per day within one week prior to the CE.

*Differences between endoscopic treatment (+) and (-) were calculated by Fisher's exact test or unpaired student t-test.

### Association between the rebleeding rate and endoscopic treatment

**Figure 3:**
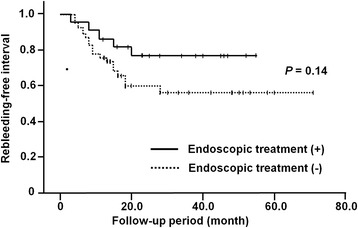
**Cumulative rebleeding rates according to the therapeutic choice.** The risk of rebleeding tended to be lower in the patients who had undergone endoscopic treatment than in those who had not received endoscopic treatment, although the difference did not reach statistical significance (*P* = 0.14, log rank test).

The overall rebleeding rate over a median follow-up duration of 30.5 months was 33.8% (23/68 cases) (interquartile range 16.5–47.0). In most cases, the first rebleeding episode occurred within 24 months after the CE, with a median time to rebleeding of 9.0 months (range 3.0–28.0). Although the rebleeding rate in the patients who had undergone endoscopic treatment was slightly lower than that in the patients who had not undergone endoscopic treatment, the difference did not reach statistical significance (22.7% vs. 39.1%, *P* = 0.27). As shown in Figure 3, the cumulative risk of rebleeding tended to be lower in the patients who had undergone endoscopic treatment than in those who had not undergone endoscopic treatment, however, this difference also did not reach statistical significance (*P* = 0.14).

### Treatment after rebleeding

The clinical outcomes after rebleeding in the patients with small bowel angioectasia are shown in Table 2. Of the 23 patients who developed rebleeding, the bleeding was overt (hematochezia or melena) in 12 (52.2%) and occult (drop of hemoglobin by more than 2 g/dl) in 11 (47.8%) patients. Eight of the 23 (34.8%) patients received additional endoscopic treatment. In the majority of the patients with rebleeding (18/23, 78.3%), the hemoglobin level increased significantly by the end of the follow-up period (9.1 ± 2.4 g/dl to 11.4 ± 2.1 g/dl, *P* <0.001).

**Table 2:** **Clinical outcomes after rebleeding in patients with small bowel angioectasia**

	**Patients with rebleeding**
Number	23
Bleeding pattern (overt/occult)	12/11
Blood transfusion, number (%)	11 (47.8)
Minimum hemoglobin value after rebleeding, g/dl	9.1 ± 2.4
Endoscopic treatment, number (%)	8 (34.8)
Controlled by the end of the follow-up period*, number (%)	18 (78.3)
Iron replacement therapy after rebleeding, number (%)	23 (100)
Hemoglobin value at the end of the follow-up period, g/dl	11.4 ± 2.1

*We defined OGIB as controlled if there was no further overt bleeding within 6 months and the hemoglobin level did not drop below 10 g/dl by the time of the final examination.

### Location of the small bowel angioectasia

A total of 239 small bowel angioectasias were identified in this study (Table 3). The lesions were multiple in 83.8% (57/68 cases) of the patients, and were present in both the proximal and the distal bowel in 42.6% (29/68 cases). The angioectasias occurred more frequently in the proximal small bowel than in the distal small bowel (64.0% vs. 36.0%). Therefore, antegrade SBE was performed more frequently to treat the small bowel angioectasia (35.2% vs. 23.2%).

**Table 3:** **Location of small bowel angioectasias**

	**Proximal**	**Distal**
Total number	153	86
Prevalence, number (%)	54 (79.4)	43 (63.2)
Endoscopic treatment, number (%)	19 (35.2)	10 (23.2)
Rebleeding, number (%)	21 (38.9)	18 (41.8)

NOTE: Each of the CE videos was divided into two segments of equal length according to the small-bowel transit time. The first segment was considered as representing the proximal small bowel and the second as representing the distal small bowel.

### Factors predicting rebleeding in patients with small bowel angioectasia

The selected variables and results are shown in Table 4. Univariate Cox proportional hazard regression analysis conducted in patients with small bowel angioectasia identified a past history of blood transfusion (odds ratio [OR] 3.16; 95% confidence interval [CI] 1.30–7.69, *P* = 0.01), presence of multiple lesions (≥3) (OR 4.31; 95% CI 1.60–11.6, *P* = 0.004), chronic kidney disease (CKD) ≥ stage 4 (OR 2.94; 95% CI 1.29–6.71, *P* = 0.01) and a history of warfarin use (OR 3.30; 95% CI 1.29–8.40, *P* = 0.01) as significant factors predictive of rebleeding. Multivariate Cox proportional hazards regression analysis identified only presence of multiple lesions (≥3) (OR 3.82; 95% CI 1.30–11.3, *P* = 0.02) as an independent significant factor predictive of rebleeding.

**Table 4:** **Predictors of rebleeding in patients with small bowel angioectasia**

**Variables**	**Rebleeding**			
	**Univariate (OR 95% CI)**	***P*** **value**	**Multivariate (OR 95% CI)**	***P*** **value**
Age >65 years	1.38 (0.54-3.52)	0.50		
Male sex	2.11 (0.87-5.15)	0.10	2.52 (0.95-6.70)	0.07
Overt bleeding	2.00 (0.79-5.08)	0.14		
Blood transfusion	3.16 (1.30-7.69)	0.01	1.08 (0.32-3.63)	0.91
Minimum hemoglobin value <8 g/dl	2.03 (0.89-4.60)	0.09	2.43 (0.95-6.19)	0.06
Size of angioectasia ≥1 mm	0.80 (0.36-1.75)	0.57		
Number of angioectasias ≥3	4.31 (1.60-11.6)	0.004	3.82 (1.30-11.3)	0.02
Drinking history	1.72 (0.75-3.95)	0.20		
Smoking history	1.71 (0.75-3.87)	0.20		
Comorbidity				
Hypertension	1.63 (0.64-4.17)	0.30		
Diabetes	0.95 (0.35-2.55)	0.92		
Cardiovascular disease	1.89 (0.80-4.48)	0.15		
Cerebral infarction	1.68 (0.57-4.95)	0.35		
CKD stage ≥4	2.94 (1.29-6.71)	0.01	1.72 (0.58-5.06)	0.33
Liver cirrhosis	3.77 (0.87-16.3)	0.08	3.44 (0.60-19.8)	0.17
Medication use				
Warfarin	3.30 (1.29-8.40)	0.01	2.48 (0.79-7.79)	0.12
LDA	1.00 (0.43-2.31)	>0.99		
Thienopyridine	1.99 (0.74-5.38)	0.17		
NSAIDs	1.32 (0.39-4.46)	0.65		
H2-blockers	0.75 (0.28-2.01)	0.56		
PPIs	1.45 (0.64-3.32)	0.38		
Rebamipide	1.17 (0.40-3.45)	0.77		

NOTE: For the multivariate Cox proportional hazard regression analysis, only the variables that were identified by univariate analysis as being significant with a P value of <0.1 were included as covariates.

*Abbreviations*: *OR*, odds ratio; *CI*, confidence interval; *CKD*, chronic kidney disease; *LDA*, low-dose aspirin; *NSAIDs*, nonsteroidal anti-inflammatory drugs; *H2-blockers*, histamine H2 receptor antagonists; *PPIs*, proton pump inhibitors.

Variable definitions: Alcohol history was defined as positive if the subject’s alcohol consumption exceeded 20 g/day. Smoking history was defined as positive if the subject had smoked more than 10-pack years and was still smoking or had quit within the previous 10 years. History of antiplatelet drug and/or NSAID use was defined as positive if the patient had been taking at least 1 pill per day of either of these drugs for more than 1 week within 1 month prior to the CE. History of anticoagulant drug use was defined as positive if the patient had been taking at least 1 pill of anticoagulant drug per day within one week prior to the CE.

## Discussion

Herein, we have presented the results of a relatively large, long-term, cohort study on bleeding from small bowel angioectasia. Our aim was to evaluate the significance of endoscopic treatment and determine the long-term outcomes in patients with small bowel angioectasia. It is worthy of note that in contrast to the case in previous studies [23-26], we excluded patients with other vascular lesions such as Dieulafoy’s lesions, varices and anteriovenous malformations from the analyses, so as to obtain important information on the optimal management of bleeding from small bowel angioectasia.

There are several reports of studies carried out to evaluate the outcomes of patients with OGIB after the initial episode of bleeding. According to these reports, the long-term rebleeding rates ranged widely from 17% to 40% [23-26,28-31]. This could be explained by the heterogeneity in the study population and differences in the duration of follow-up, management strategies and definition of rebleeding among studies. Importantly, the reported rebleeding rates associated with vascular lesions are higher than those associated with other lesions, such as erosions/ulcers and tumors [23-26]. Consistent with previous reports, we confirmed a relatively high rebleeding rate (33.8%) in patients with small bowel angioectasia.

Our results indicated that initial endoscopic treatment was not sufficient to control the risk of rebleeding from small bowel angioectasia. This might be attributable to the following reasons. Firstly, in some patients, even if the lesions thought to be responsible for the bleeding are treated appropriately, other tiny lesions can bleed later. In this study, only 5 patients (including 4 with bleeding from proximal angioectasia and 1 with bleeding from distal angioectasia) were identified as having ongoing active bleeding by CE examination. Most angioectasias are reported to be non-incidental lesions [32]. Thus it is difficult to identify which the angioectasia is the source of the bleeding. Consistent with a previous report [32], multiple lesions were observed at a high frequency in this study (83.8%), and 42.6% of the patients had angioectasias both in the proximal and in the distal small bowel. Therefore, all of the lesions, especially some of the tiny lesions, could not be treated in a single endoscopic treatment session, even though an attempt was made to treat all the definitive lesions. In the present study, the size of the angioectasia was not found to be a factor associated with rebleeding. Moreover, Shiozaki et al. reported that tiny lesions rather than larger lesions bled more frequently [25]. These results suggest that aggressive endoscopic treatment of tiny lesions is important to prevent future rebleeding. In addition, careful follow-up is required when only tiny lesions are detected as the bleeding source. Secondly, definitive lesions could be overlooked by the first CE examination in patients with OGIB. Fujimori et al. reported that some angioectasias could be diagnosed only later even after combined CE and double-balloon enteroscopy (DBE) examination [33]. Small bowel angioectasia is reported to occur more frequently in the proximal small bowel than in the distal small bowel [23,34]. Because of rapid capsule transit or reduced bowel visibility due to the presence of bile and bubble artifacts [35], small bowel lesions, especially those located in the proximal small bowel, are likely to be overlooked by CE. Recently, improved visibility and detectability of small bowel angioectasia has been reported with the use of computed virtual chromoendoscopy systems, such as flexible spectral imaging color enhancement (FICE) [36,37]. Additional studies are needed to evaluate whether the use of such image-enhancing modalities could contribute to reducing the risk of rebleeding from small bowel angioectasia. Finally, the lower diagnostic yield of SBE could have affected the outcomes of the patients enrolled in this study. SBE was subsequently introduced to avoid the time-consuming and complex DBE procedure. Although SBE and DBE appear to be equivalent in terms of the therapeutic outcomes and re-bleeding rates for small bowel lesions [38,39], decreased rates of total enteroscopy was observed in cases examined by SBE.

Consistent with a previous report [25], presence of multiple lesions was identified in this study as a significant risk factor for the occurrence of rebleeding. In the present study, while the first rebleeding episode occurred after 1 year in approximately 50% of the cases, in the majority of cases, rebleeding occurred within 2 years, suggesting that patients with small bowel angioectasia, especially those with multiple lesions, should be closely followed up for at least 2 years after the initial treatment. Although our results suggested that rebleeding could be controlled by repeat endoscopic treatment and iron replacement therapy in the majority of patients with small bowel angioectasia, some patients may be unsuitable for endoscopic treatment, as angioectasia is frequently detected in patients older than 60 years of age [32] and is often accompanied by severe comorbidities such as chronic renal failure [40] and cardiac valvular disease [41]. Pharmacological treatments such as thalidomide and octoreotide might serve as attractive options for these patients [42,43]. Use of anticoagulant therapy was not identified as an independent significant predictor of rebleeding. In this study, only 9 patients were receiving anticoagulant therapy, therefore, the small sample size could have affected the results of this study.

The present study had some limitations. Firstly, it was a retrospective study, therefore, a selection bias was inevitable. Secondly, the number of patients enrolled in this study was not sufficiently large, which could have limited our conclusions. Thirdly, not all patients underwent subsequent BAE for confirmation of the results of CE. Finally, the choice of treatment and follow-up procedures were not selected based on a randomized controlled trial protocol. To establish medical management of bleeding from small bowel angioectasia, a large prospective randomized controlled trial is needed.

## Conclusions

Our results indicate that OGIB patients with small bowel angioectasia show relatively high rebleeding rates. Although a single session of endoscopic treatment was not sufficient to prevent rebleeding in the future, in most cases, rebleeding could be controlled with repeat endoscopic treatment and/or iron replacement therapy. Careful follow-up is needed for patients with multiple lesions, presence of which was identified as a significant risk factor for rebleeding.

## Abbreviations

CE: Capsule endoscopy
OGIB: Oscure gastrointestinal bleeding
BAE: Single-balloon enteroscopy
SBE: Balloon-assisted enteroscopy
DBE: Double-balloon enteroscopy
APC: Argon plasma coagulation
IQR: Interquartile range
OR: Odds ratio
CI: Confidence interval
CKD: Chronic kidney disease
LDA: Low-dose aspirin
NSAIDs: Nonsteroidal anti-inflammatory drugs
H2-blockers: Histamine H2 receptor antagonists
PPIs: Proton pump inhibitors
FICE: Flexible spectral imaging color enhancement
